# Glycemic control by the SGLT2 inhibitor empagliflozin decreases aortic stiffness, renal resistivity index and kidney injury

**DOI:** 10.1186/s12933-018-0750-8

**Published:** 2018-07-30

**Authors:** Annayya R. Aroor, Nitin A. Das, Andrea J. Carpenter, Javad Habibi, Guanghong Jia, Francisco I. Ramirez-Perez, Luis Martinez-Lemus, Camila M. Manrique-Acevedo, Melvin R. Hayden, Cornel Duta, Ravi Nistala, Eric Mayoux, Jaume Padilla, Bysani Chandrasekar, Vincent G. DeMarco

**Affiliations:** 1 Diabetes and Cardiovascular Center, University of Missouri School of Medicine, Columbia, MO USA; 2 Division of Endocrinology and Metabolism, Department of Medicine, University of Missouri, Columbia, MO USA; 3 Research Service, Harry S. Truman Memorial Veterans Hospital, Columbia, MO USA; 4 Division of Nephrology, Department of Medicine, University of Missouri, Columbia, MO USA; 5 Division of Cardiology, Department of Medicine, University of Missouri, Columbia, MO USA; 6 Cardiothoracic Surgery, University of Texas Health Science Center, San Antonio, San Antonio, TX USA; 7 Boehringer Ingelheim, Biberach an der Riss, Germany; 8 Department of Nutrition and Exercise Physiology, University of Missouri, Columbia, MO USA; 9 Department of Child Health, University of Missouri, Columbia, MO USA; 10 Dalton Cardiovascular Research Center, University of Missouri, Columbia, MO USA; 11 Department of Medical Pharmacology and Physiology, University of Missouri, Columbia, MO USA; 12 Division of Endocrinology and Metabolism, Department of Medicine, University of Missouri-Columbia School of Medicine, D110, DC043.0, One Hospital Dr, Columbia, MO 65212 USA

**Keywords:** Vascular stiffness, Renal resistivity, SGLT2, RECK, Pulsatility index

## Abstract

**Background:**

Arterial stiffness is emerging as an independent risk factor for the development of chronic kidney disease. The sodium glucose co-transporter 2 (SGLT2) inhibitors, which lower serum glucose by inhibiting SGLT2-mediated glucose reabsorption in renal proximal tubules, have shown promise in reducing arterial stiffness and the risk of cardiovascular and kidney disease in individuals with type 2 diabetes mellitus. Since hyperglycemia contributes to arterial stiffness, we hypothesized that the SGLT2 inhibitor empagliflozin (EMPA) would improve endothelial function, reduce aortic stiffness, and attenuate kidney disease by lowering hyperglycemia in type 2 diabetic female mice (db/db).

**Materials/methods:**

Ten-week-old female wild-type control (C57BLKS/J) and db/db (BKS.Cg-Dock7m+/+Leprdb/J) mice were divided into three groups: lean untreated controls (CkC, n = 17), untreated db/db (DbC, n = 19) and EMPA-treated db/db mice (DbE, n = 19). EMPA was mixed with normal mouse chow at a concentration to deliver 10 mg kg^−1^ day^−1^, and fed for 5 weeks, initiated at 11 weeks of age.

**Results:**

Compared to CkC, DbC showed increased glucose levels, blood pressure, aortic and endothelial cell stiffness, and impaired endothelium-dependent vasorelaxation. Furthermore, DbC exhibited impaired activation of endothelial nitric oxide synthase, increased renal resistivity and pulsatility indexes, enhanced renal expression of advanced glycation end products, and periarterial and tubulointerstitial fibrosis. EMPA promoted glycosuria and blunted these vascular and renal impairments, without affecting increases in blood pressure. In addition, expression of “reversion inducing cysteine rich protein with Kazal motifs” (RECK), an anti-fibrotic mediator, was significantly suppressed in DbC kidneys and partially restored by EMPA. Confirming the in vivo data, EMPA reversed high glucose-induced RECK suppression in human proximal tubule cells.

**Conclusions:**

Empagliflozin ameliorates kidney injury in type 2 diabetic female mice by promoting glycosuria, and possibly by reducing systemic and renal artery stiffness, and reversing RECK suppression.

## Introduction

Diabetic kidney disease (DKD) is the most common cause of end stage renal disease and the mechanisms underlying its development are complex and still not well understood [1–3]. In this regard, arterial stiffness is an independent and robust predictor of chronic kidney and cardiovascular diseases [4, 5]. Measurement of aortic pulse wave velocity (PWV) is the reference standard technique for assessment of central aortic stiffness [4–6]. Although aortic stiffness is considered a disease of aging, stiffening is accelerated during obesity and diabetes, and can be present at earlier ages [7]. Additionally, renal microvasculature stiffening, indicated by increased renal resistivity index (RRI), is associated with albuminuria, thereby suggesting a role for stiffening of both the macro- and micro-vasculature in the development of kidney injury [5, 7–9]. Indeed, accumulating evidence indicates that increased PWV predicts adverse macrovascular and microvascular outcomes in diabetics with albuminuria and kidney disease [4, 5, 10]. In addition, renal tubulointerstitial fibrosis also contributes to renal microvascular stiffness, underscoring the intricate relationship between renal pathology and microvascular complications [6, 7, 11].

Current drugs targeting progression of DKD include anti-hyperglycemic agents, and inhibitors of inappropriate RAAS (renin–angiotensin–aldosterone system) activation, hypertension and hyperlipidemia [3]. Despite widespread use of these therapeutics, more than 30% of patients with type 2 diabetes mellitus (T2DM) develop DKD [3]. Recently, inhibition of the sodium-glucose co-transporter 2 (SGLT2) in kidneys, a more recent and novel therapeutic approach to lower hyperglycemia, has been shown to slow progression of DKD in preclinical models and clinical studies, either as stand-alone or combination therapy, including DPP-4 inhibitors, GLP-1 receptor antagonist, or ACEi/ARBs [12–14]; however the mechanisms are not entirely known [15–21]. SGLT2 inhibitors induce glycosuria by reducing reabsorption of glucose in proximal tubules, and evidence from clinical trials indicates that they also exert cardiovascular protection [17–26]. Indeed, in a phase III clinical trial, empagliflozin (EMPA), a potent and highly selective SGLT2 inhibitor [27], was shown to reduce blood pressure (BP), but also markers of arterial stiffness [28]. EMPA-mediated improvements in central hemodynamics, vascular function and arterial stiffness were further corroborated in a recent double-blind, randomized, placebo-controlled crossover, interventional study involving 76 T2DM patients [29]. However, its effects on renal fibrosis, the *sine qua non* of chronic kidney disease, are not fully understood.

Several studies support a role for reversion-inducing-cysteine-rich protein with Kazal motifs (RECK), a membrane-anchored matrix metalloproteinase (MMP) regulator, in suppressing pro-fibrotic responses through inhibition of MMP activation [30, 31]. Although the role of RECK deficiency in promoting angiogenesis [32] and cardiac fibrosis [30] has been reported previously, the influence of low RECK expression on renal injury and fibrosis in diabetes has not been investigated. Therefore, we hypothesized that SGLT2 inhibition by EMPA blunts diabetic kidney injury and fibrosis by suppressing macrovascular/microvascular stiffness and upregulating anti-fibrotic RECK expression in the kidney. We further posited that the renovascular protective effects of EMPA are attributable to improved glycemic control, enhanced activation of endothelial nitric oxide synthase (eNOS), and suppressed oxidative stress. Herein we report that improved glycemic control by EMPA ameliorates kidney injury in female diabetic (db/db) mice by reducing systemic and renal artery stiffness, and restoring RECK expression.

## Methods

### Animals and treatments

Animal studies were approved by the Institutional Animal Care and Use Committees at Harry S Truman Memorial Veterans’ Hospital and University of Missouri, Columbia, MO, and conform to NIH guidelines. Eight-week-old female db/db (BKS.Cg-Dock7m+/+Leprdb/J) and wild-type control (C57BLKS/J) mice were purchased from The Jackson Laboratory (Bar Harbor, ME) and housed under standard laboratory conditions where room temperature was 21–22 °C and light and dark cycles were 12 h each. Three different cohorts of mice were used: lean untreated controls (CkC, n = 17), untreated db/db (DbC, n = 19) and EMPA-treated db/db mice (DbE, n = 19) for 5 weeks, initiated at 11 weeks of age. It should be noted that prior to treatment start, 10 week old db/db mice were weighed and assigned to DbC or DbE groups so that the mean weight of each group was similar. EMPA was mixed with normal mouse chow (Purina Diet 5008; Test Diet^®^, Richmond, IN) at a concentration of 60 mg kg^−1^ of diet calculated to deliver 10 mg kg^−1^ day^−1^ based on food intake [33]. This dose improves HbA1c, blood glucose levels and insulin sensitivity in db/db mice [33]. Purina diet 5008, the most common lab diet formulation used to feed mice, contains 0.28% sodium.

### Urine analysis

Two to three days before study end, mice were placed in metabolic chambers for 24-h urine collection. Urine was analyzed for microalbumin, creatinine, and microalbumin/creatinine ratio using a DCA Vantage analyzer (Siemens, Malvern, PA), according to manufacturer’s instructions.

### In vivo aortic stiffness by PWV and ex vivo endothelial cell (EC) stiffness by atomic force microscopy (AFM)

At study end, in vivo aortic stiffness was evaluated in isoflurane-anesthetized mice (1.75% in 100% oxygen stream) by PWV using Doppler ultrasound (Indus Mouse Doppler System, Webster, TX) [34, 35]. Briefly, the transit time method was utilized to determine PWV which is calculated as the difference in arrival times of a Doppler pulse wave at two locations along the aorta at a fixed distance [36]. Pulse wave arrival times are measured as the time from the peak of the ECG R-wave to the leading foot of the pulse wave at which time velocity begins to rise at the start of systole. The distance between the two locations along the aorta is divided by the difference in arrival times and is expressed in m/s. Velocity waveforms were acquired at the aortic arch followed immediately by measurement at the descending aorta 35 mm distal to the aortic arch.

The stiffness of EC, measured as the force exerted by a stylus probe on the luminal surface of aortic explants, was measured by a nano-indentation technique utilizing AFM, as previously described by us [34]. Briefly, a 2 mm ring of the thoracic aorta was isolated from mice following the 5-week experimental period to assess the stiffness of EC. The aortic ring was opened longitudinally and the adventitial surface of each explant was fastened to a glass cover slip using Cell-tak so that we had en face access to the EC surface for placement of the AFM stylus. Stiffness of the EC surface was estimated by placing the stylus at approximately 15 random locations along the EC surface of an explant and determining the average EC stiffness for that aorta.

### Ex vivo wire myography of aorta

Aortic vasomotor responses were examined as previously described [34, 37]. Briefly, a 2 mm segment of thoracic aorta was collected immediately after euthanasia and placed in ice-cold physiological salt solution (PSS) containing (in mM): 119 NaCl, 4.7 KCl, 2.5 CaCl, 1.18 KH_2_PO_4_, 1.17 MgSO_4_, 0.027 EDTA, 5.5 glucose, and 25 NaHCO_3_, pH 7.4. Aortic contractile state was ascertained by KCl (80 mM). Aortas were preconstricted with U46619 (100 nM). Relaxation response of arterial rings to acetylcholine (1 nM to 100 μM) was assessed by cumulative addition of agonist to the vessel bath. At the end of each experiment, the PSS bath solution was replaced with Ca^2+^-free PSS to determine maximal passive diameter. Aortic dilator responses are presented as percent maximal relaxation.

### Ultrastructure analysis using transmission electron microscopy (TEM)

Briefly, 1 mm segments of thoracic aortic rings were placed in TEM fixative, embedded and sectioned, as previously described [37]. Images were captured using a JOEL 1400-EX TEM (Joel, Tokyo, Japan). Three fields were randomly chosen per mouse to obtain three 2000× images per aorta. The lengths of three ECs were measured in samples from four mice in each group.

### Renal artery indices of vascular dysfunction

B-mode and Pulsed-Wave Doppler ultrasound was performed by a single experienced observer in a blinded fashion using a Vevo 2100 (FUJIFILM VisualSonics, Ontario, Canada) for measurement of RRI and pulsatility index (PI). Images of the left renal artery (LRA) were acquired in Color Doppler mode to visualize arterial blood flow. A sample volume was placed in the left renal artery just proximal to the kidney pelvis and pulse-wave spectra were captured and analyzed offline to acquire peak systolic velocity (PSV), lowest diastolic velocity (LDV) and mean velocity (MV) determined from the velocity time integral. Parameters were measured in triplicate using three separate spectra. The resistive index (RI) was calculated as: RI = (PSV − LDV)/PSV. The Pulsatility Index (PI) was calculated as: PI = (PSV − LDV)/MV.

### Renal artery stiffness and structural remodeling

Proximal renal arteries were cannulated, pressurized at 70 mmHg and warmed to 37 °C in myograph chambers (Living Systems Instrumentation, Burlington, VT, USA). Elastic properties were characterized under passive conditions. Arteries were exposed to step-wise increments in intraluminal pressure. Luminal diameter (*D*) and wall thicknesses ($\tau$) were recorded, and mechanical properties were calculated as previously described [38]. The calculated incremental pulse wave velocity ($cPW{V_{inc}}$) is the value of the PWV of the renal arteries as obtained using the Moens–Korteweg equation [39]:

$$cPW{V_{inc}} = \sqrt {\frac{{{E_{inc}} \cdot \tau }}{\rho \cdot D}} ,$$

the incremental modulus of elasticity (${E_{inc}}$), *D* and $\tau$ were measured for a given intraluminal pressure and $\rho$ represents the density of the intraluminal buffer ($\rho \approx 1005\;\;{\text{kg}}/{{\text{m}}^3}$) used in the experiments.

### Quantification of kidney fibrosis

At the end of the study, two mm thick slices of kidney were fixed in paraformaldehyde (PFA), embedded in paraffin, sectioned at five microns and stained for collagens using picro-Sirius red (PSR), as previously described [40]. Briefly, for each animal an average estimate of periarterial fibrosis was determined by quantifying PSR staining in the adventitia surrounding three randomly selected cortical arterioles. In addition, we estimated average peritubular fibrosis for each mouse by quantifying PSR stain within two randomly selected low magnification (4×) regions of interest rectangles in the cortex of the kidney.

### Immunohistochemistry/immunofluorescence

PFA fixed, paraffin embedded five micron thick kidney sections were incubated with primary antibodies to 3-nitrotyrosine (1:150, # AB5411; Millipore, Billerica, MA, USA), advanced glycation end products AGE (1:100, #23722, Abcam), or RECK (1:50, # 3433, Cell Signaling Technology, Inc) as previously described [40, 41]. For visualization of immunofluorescence signals the following secondary antibodies were used; biotinylated link and streptavidin HRP for 3-NT (Dako, Carpiteria, CA) and Alexa Flour 647 donkey anti rabbit 1:300 in tenfold diluted blocker for both AGE and RECK (Invitrogen). Images were captured using a Leica multiphoton confocal microscope. The fluorescence intensity was measured using MetaMorph software and presented as average grey scale intensities.

### Immunoblotting

Preparation of whole cell homogenates, immunoblotting, detection of the immunoreactive bands by enhanced chemiluminescence, and quantification by densitometry were performed as described previously [30]. Briefly, kidney homogenates were separated on a 4–15% precast gel, electrophoresed at 100 V. Separated proteins were transferred onto PVDF. After blocking, membranes were incubated with RECK (1:1000; catalog# 3433, Cell Signaling Technology, Inc), phospho-eNOS^Ser1177^ (1:1000; catalog# 92705, Cell Signaling Technology, Inc) and phospho-eNOS^Thr495^ (1:1000; catalog# 95745, Cell Signaling Technology, Inc), collagen Iα1 (1 μg/ml; catalog# ab34170, abcam), collagen IIIα1 (1 μg/ml; catalog# ab7778, abcam), or fibronectin (1 μg/ml; catalog# ab23750, abcam) for 1 h at room temperature. GAPDH (1:1000, catalog# sc-25779, Santa Cruz Biotechnology, Inc) served as an invariant control. Washed membranes were incubated with a secondary antibody (1:3000, anti-Rabbit IgG HRP linked antibody, catalog# 7074S, Cell Signaling Technology, Inc.) for 1 h at room temperature. Following a 15 s exposure in a gel doc imager, the intensities of immunoreactive bands were quantified by NIH Image J software. The results were normalized to corresponding GAPDH, and presented as a fold change.

### Culture of human proximal tubular epithelial cells

Human proximal tubular epithelial cells (HK-2, CRL-2190TM, ATCC, Manassas, VA) were cultured in Dulbecco’s modified Eagle’s medium (DMEM)/F-12 medium supplemented with 10% heat-inactivated FBS and penicillin–streptomycin in a humidified atmosphere containing 5% CO_2_ at 37 °C. At 70% confluency, complete medium was replaced with medium containing 0.5% BSA for 16 h. These serum-starved cells were then incubated with 25 mM d-glucose (high glucose). l-Glucose (l-glucose 20 mM + d-glucose 5 mM) served as a control. Mannitol (mannitol, 20 mM + d-glucose, 5 mM) served as an osmolar control. After a 6 h incubation, cells were harvested, proteins extracted and extracts were analyzed for RECK expression by immunoblotting using anti-RECK antibody. GAPDH served as a loading control. In a subset of experiments, the serum-starved HK-2 cells were incubated with EMPA (500 nM in DMSO for 15 min) prior to the addition of high glucose for 6 h. DMSO served as solvent control, and its levels did not exceed 0.1%. Neither HG, DMSO nor EMPA affected cell viability as analyzed by cell viability assay (Vybrant^®^ MTT Cell Proliferation Assay Kit, Thermo Fisher Scientific; data not shown). Representative images from three independent immunoblotting experiments and densitometry results are shown.

### Statistical analysis

Results are reported as the mean ± SE. Differences in outcomes were determined using one-way ANOVA and Bonferroni post hoc tests for paired comparisons and were considered significant when p < 0.05. Comparisons of pre- and end-treatment PWV for DbC and DbE were analyzed using two-way repeated measures ANOVA and post hoc pairwise comparisons were analyzed using the Bonferroni test. The main effects were treatment (EMPA vs. no EMPA) and time (pre versus end) and the interaction of treatment and time. Relations between aorta and kidney functional parameters were assessed using linear regression analysis. All statistical analyses were performed using Sigma Plot (version 12) software (SYSTAT Software, Point Richmond, CA).

## Results

### Baseline parameters

We previously reported baseline metabolic parameters and 24-h ambulatory systolic (SBP) and diastolic BP (DBP) measurements in the same cohort of mice utilized in the present study [40]. Fasting blood glucose concentrations prior to initiation of EMPA treatment were 8.6 ± 0.8, 15.8 ± 2.5 and 21.4 ± 2.7 mmol/l, respectively for CkC, DbC and DbE (p < 0.05 for CkC vs. DbC and CkC vs. DbE; p > 0.05 for DbC vs. DbE), indicating hyperglycemia in both DbC and DbE. At the end of the study, fasting blood glucose concentrations were 7.7 ± 0.8, 29.3 ± 2.4 and 14.6 ± 1.8 mmol/l, respectively (p < 0.05 for CkC vs. DbC and CkC vs. DbE; p < 0.0001 for DbC vs. DbE), indicating hyperglycemia worsened in DbC and improved in DbE. Compared to CkC, DbC exhibited increases in whole body fat mass (12-fold), plasma cholesterol and triglycerides, glycemic indexes (e.g., HbA1c and fasting glucose), and SBP and DBP. Although EMPA significantly reduced HbA1c and fasting glucose levels, it did not lower SBP, DBP, fat mass, cholesterol or triglycerides. Further, compared to CkC, DbC and DbE exhibited 54- and 111-fold increases in urine glucose excretion, respectively [40].

### EMPA reduces aortic and EC stiffness, RRI and albuminuria

**Fig. 1:**
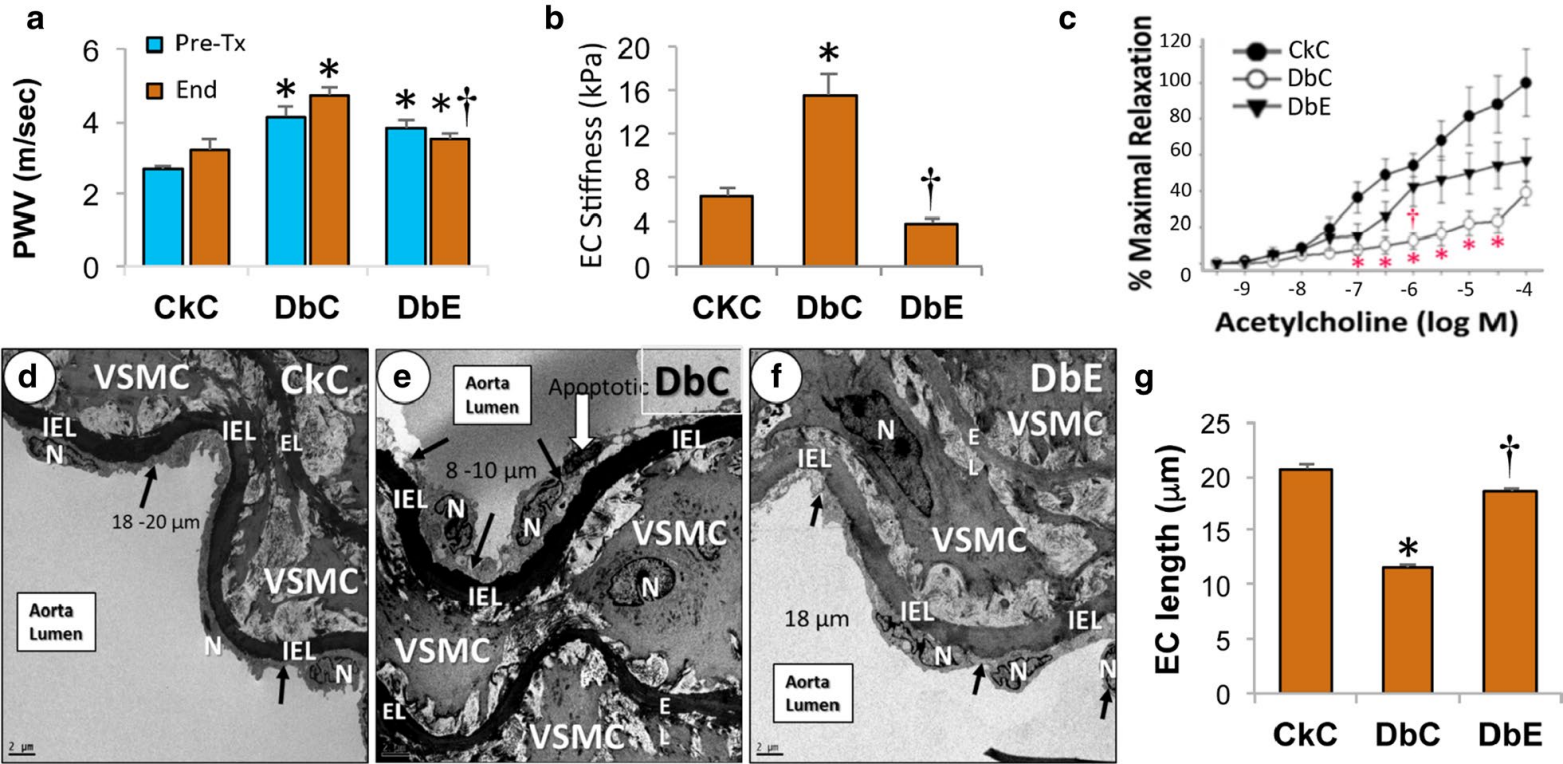
Empagliflozin improves aortic stiffness. **a** EMPA ameliorates progression of aortic stiffening, assessed by in vivo measures of pulse wave velocity (PWV) (n = 5–6/group). **b** Aortic endothelial stiffness was evaluated in ex vivo aortic explants utilizing atomic force microscopy (n = 5–6/group). **c** Vasomotor responses to acetylcholine using ex vivo wire myography (n = 5/group). **d**–**g** Diabetic female db/db mice (DbC) exhibit shortening–contraction, separation, lifting and apoptosis of endothelial cells compared to lean control (CkC) and diabetic db/db mice treated with EMPA (DbE). **d** Illustrates that endothelial cell(s) (EC) (arrows) are elongated and tightly adherent to the internal elastic lamina in the CkC. **e** Depicts the shorter–contracted ECs in the DbC. Note the shortening–contraction and loss of elongation of the ECs. Also note the one EC appears to demonstrate separation and lifting, thinning and early apoptotic electron dense Nucleus (N) (open arrow). **f** Demonstrates that EMPA treatment (DbE) protects these EC from undergoing the remodeling changes noted in DbC and are more elongated and tightly adherent similar to CkC. A semi-quantitative analysis of EC length is shown in the bar graph (**g**) and indicates significantly shorter EC in DbC compared to CkC and DbE. The lengths (μm) of three EC were measured in samples from four mice in each group. Magnification ×800; bar = 2 µm. *EL* media elastic lamina, *VSMC* vascular smooth muscle cell. *p < 0.05 vs. CkC; ^†^p < 0.05 vs. DbC. All values are mean ± SE

Prior to beginning of the 5-week treatment period, DbC and DbE exhibited significantly higher baseline aortic PWV (vs. CkC, Fig. 1a; p < 0.05), indicative of aortic stiffness. At the end of the treatment period, DbC had slightly higher PWV compared to baseline, indicating further progression in aortic stiffening, an effect ameliorated by EMPA treatment (DbE vs. DbC, p < 0.05). Repeated measures of ANOVA indicated a significant treatment by time interaction, whereby PWV was increased over time in DbC (p < 0.05), but not DbE. Further, AFM performed on thoracic aorta explants revealed increased EC surface stiffness (kPa) relative to CkC (Fig. 1b; p < 0.05). Stiffening of EC occurred in parallel with impairments in acetylcholine-induced vasorelaxation in DbC compared to CkC (Fig. 1c, p < 0.05). Importantly, EMPA treatment abrogated both aortic EC stiffness and abnormal responses to acetylcholine (Fig. 1b and c; DbE vs. DbC, p < 0.05).

Though no histologic changes to the aorta were detected at the light microscopic level (data not shown), electron microscopy revealed evidence of ultrastructural remodeling in DbC. Specifically, aortic EC of CkC were elongated and tightly adherent to the internal elastic lamina, while those in DbC were shorter-contracted and were less adherent with some lifting or separation from the IEL (Fig. 1d and e). Also, an electron dense nucleus was observed in an abnormal EC from DbC, suggestive of early apoptosis. EMPA treatment (DbE) protected capillary EC from undergoing the remodeling changes noted in DbC (Fig. 1f). A semi-quantitative analysis of EC length (μm) indicated significantly shorter EC in DbC compared to CkC and DbE (Fig. 1g).

**Fig. 2:**
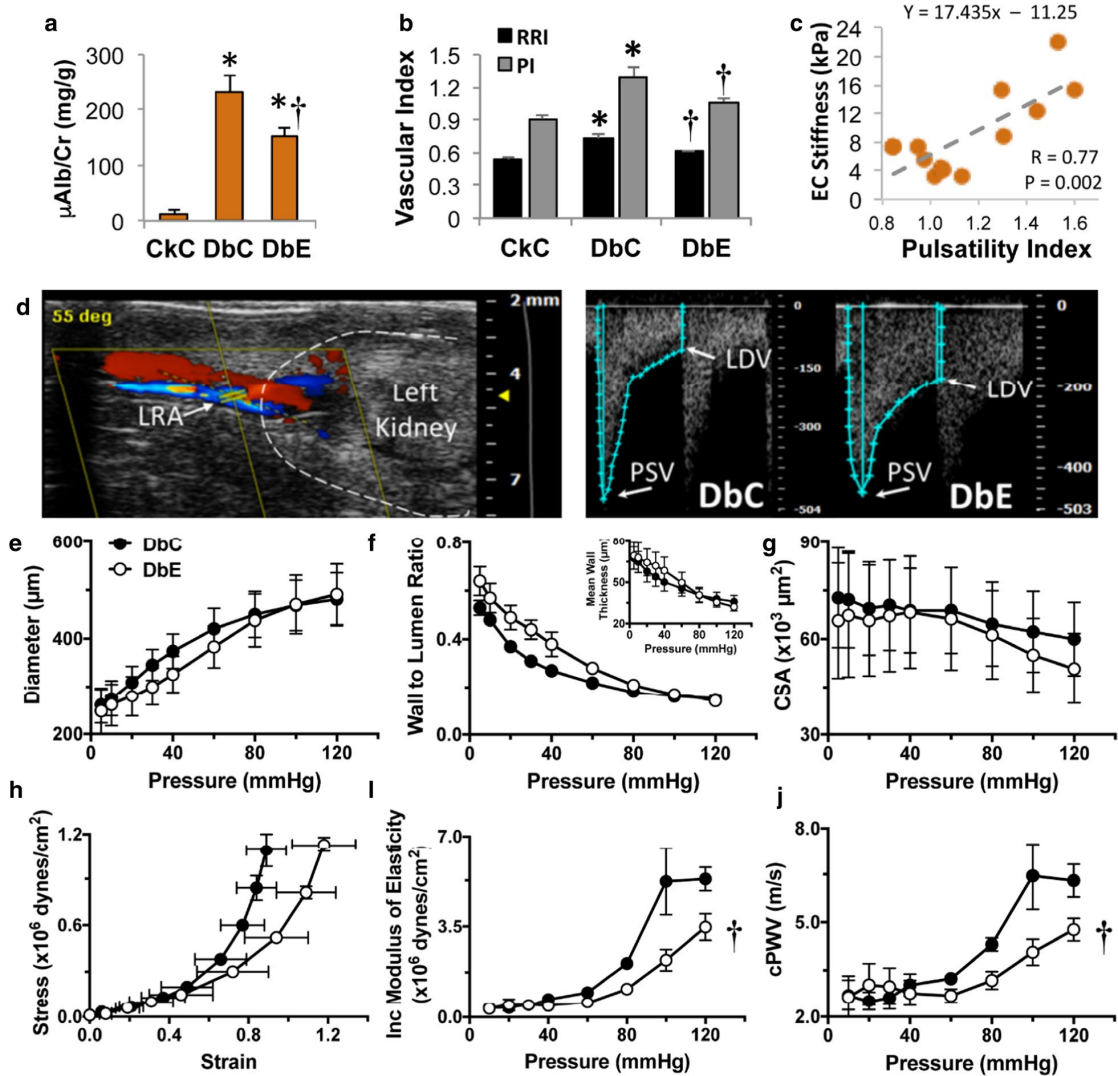
Empagliflozin ameliorates microalbuminuria and renal artery stiffness. **a** EMPA ameliorates progression of microalbuminuria and **b** renal resistivity index (RRI) and pulsatility index (PI) (N = 5–6/group). **c** Correlation analysis indicates a positive relationship between EC stiffness and PI. **d** The left side of panel shows Color Doppler flow in the left renal artery (LRA) and the right panel shows representative Doppler flow spectra obtained to calculate RRI and PI for DbC and DbE (CkC not shown). **e**–**j** Mechanical properties of renal arteries were evaluated by ex vivo pressure myography in vessels from DbE and compared with those of vessels from DbC (n = 5/group). **e** Pressure diameter curves. **f** Wall/lumen ratios and mean wall thickness (insert). **g** Wall cross-sectional area (CSA). **h** Strain–stress relationships showing that EMPA increased the distensibility of renal arteries compared to those of DbC. **i** Incremental moduli of elasticity showing that EMPA reduced the stiffness of renal arteries. **j** The calculated incremental pulse wave velocity (cPWV_*inc*_) of renal arteries from EMPA treated mice was significantly reduced compared to those from DbC. The values are mean ± SE. *PSV* peak systolic velocity, *LDV* lowest diastolic velocity, *p < 0.05 vs. CkC; ^†^p < 0.05 vs. DbC

The increase in aortic PWV in DbC and accompanying increases in EC stiffness and dysfunction were associated with increased albuminuria (e.g., an increase in microalbumin:creatinine ratio) (Fig. 2a, p < 0.05), as well as increases in RRI and PI (Fig. 2b CkC vs. DbC, p < 0.05). Notably, EMPA ameliorated these functional abnormalities in the kidney (Fig. 2a and b) (DbE vs. DbC, p < 0.05). Given these observations indicating concomitant aortic and renal vascular impairments, we next evaluated whether stiffness parameters in the aorta and kidney are correlated. Simultaneous measures of each variable were available from four mice in each of the three treatment groups. In fact, a plot of aortic endothelial stiffness and PI (Fig. 2c) demonstrated a significant positive correlation (r = 0.77; p < 0.05), indicating that increased aortic stiffness is associated with increased renal PI. A similar positive correlation (graph not shown) was observed between endothelial stiffness and RRI (EC stiffness = 39.9(RRI) − 14.5; r = 0.75; p = 0.003). Additionally, RRI was moderately correlated with aortic PWV (RRI = 0.082(PWV) + 0.329; r = 0.62; p = 0.018), as was PI (PI = 0.172(PWV) + 0.473; r = 0.62; p = 0.018) (graphs not shown).

### EMPA ameliorates renal artery stiffness

In order to determine the specific effects of EMPA treatment on the structural and mechanical characteristics of the renal circulation, we isolated and cannulated portions of the renal artery from DbC and DbE mice and subjected them to incremental changes in intraluminal pressure under passive conditions. Measurements of internal diameter and wall thickness served to calculate pressure diameter curves, wall cross-sectional areas, strain–stress relationships, incremental moduli of elasticity and cPWV_*inc*_. EMPA did not have significant effects on the pressure diameter curves, the wall to lumen ratios or the wall cross-sectional areas of the renal arteries (Fig. 2e–g). However, EMPA made renal arteries more distensible and less stiff relative to those from DbC (Fig. 2h, i), due primarily to presence of smaller diameters at lower pressures in vessels from DbE. These results were supported by the presence of reductions in the cPWV_*inc*_ of renal arteries from DbE compared with those from DbC (Fig. 2j), indicating that EMPA reduced the stiffness of the renal circulation associated with diabetes.

### EMPA reduces kidney fibrosis

**Fig. 3:**
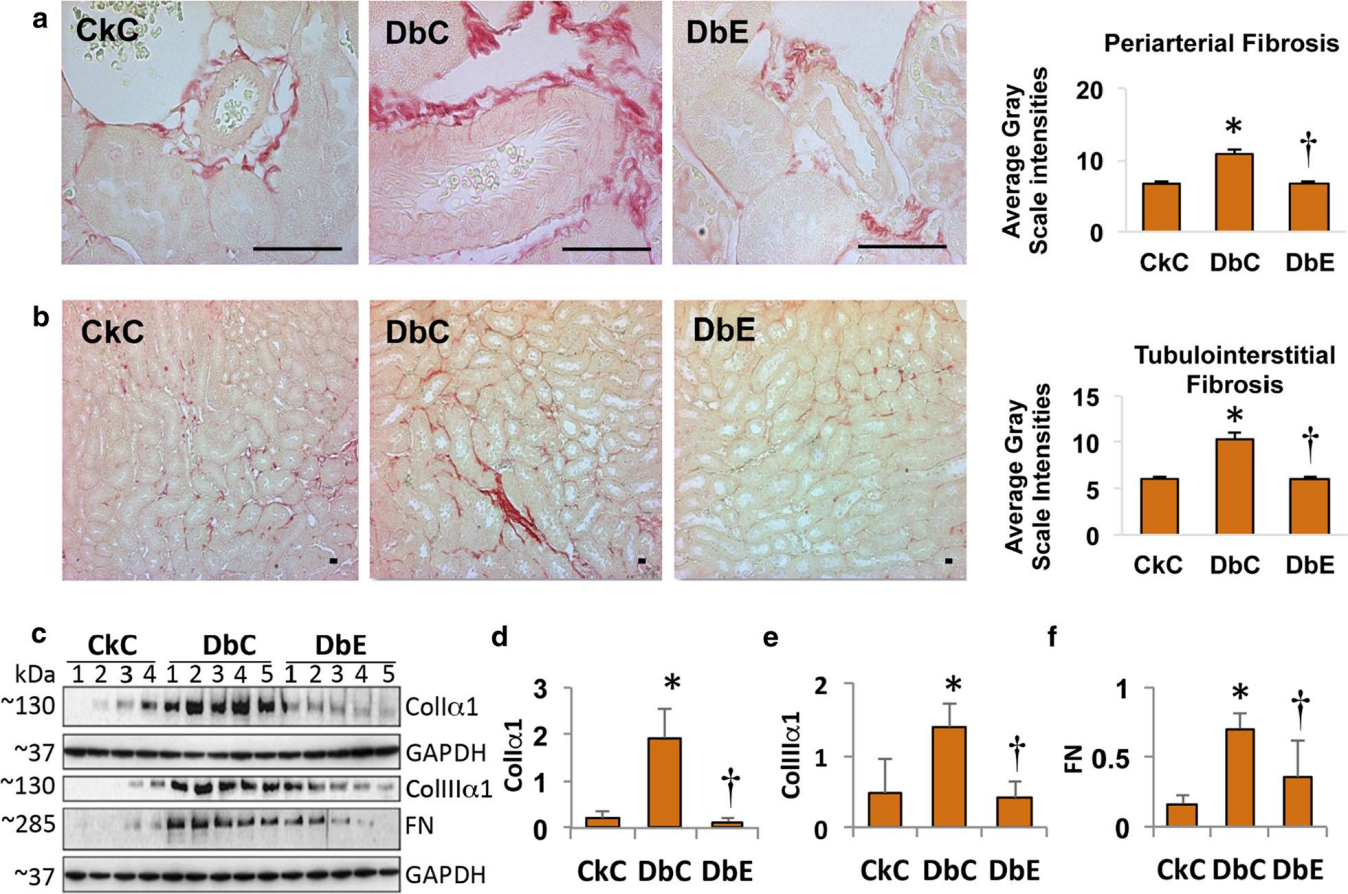
Empagliflozin ameliorates periarterial and tubulointerstitial fibrosis in the kidney. **a** Representative images show periarterial and **b** tubulointerstitial fibrosis, assessed by picrosirius red staining (PSR, magnification = 40× and 4×, respectively). The accompanying bar graphs shows semi-quantitative analysis of average PSR stain intensities obtained from five randomly selected regions of interest (n = 7 mice/group). **c** Western blot analysis shows changes in expression of collagen (Col) Iα1, Col IIIα1, FN (fibronectin) and GAPDH. The accompanying bar graphs (**d**–**f**) show quantitative analysis of protein expression normalized to GAPDH. *p < 0.05 vs. CkC; ^†^*p *< 0.05 vs. DbC. All values are mean ± SE

In relation to the observed increases in aortic (macrovascular) stiffness and RRI (microvascular stiffness) in DbC, there were increases in periarterial and interstitial fibrosis in kidney cortical tissue compared to CkC (Fig. 3a and b, p < 0.05; respectively). Contemporaneous with these changes there were also increases in collagen Iα1, IIIα1 and fibronectin (FN) protein expression in kidneys of the DbC, compared to CkC (Fig. 3c–f, p < 0.05; respectively). Notably, EMPA repressed the expression of these fibrotic markers (p < 0.05), indicating that SGLT2 inhibition by EMPA exerts anti-fibrotic effects in kidneys of diabetic mice.

### EMPA differentially regulates AGE expression, eNOS activation and nitrosative stress

**Fig. 4:**
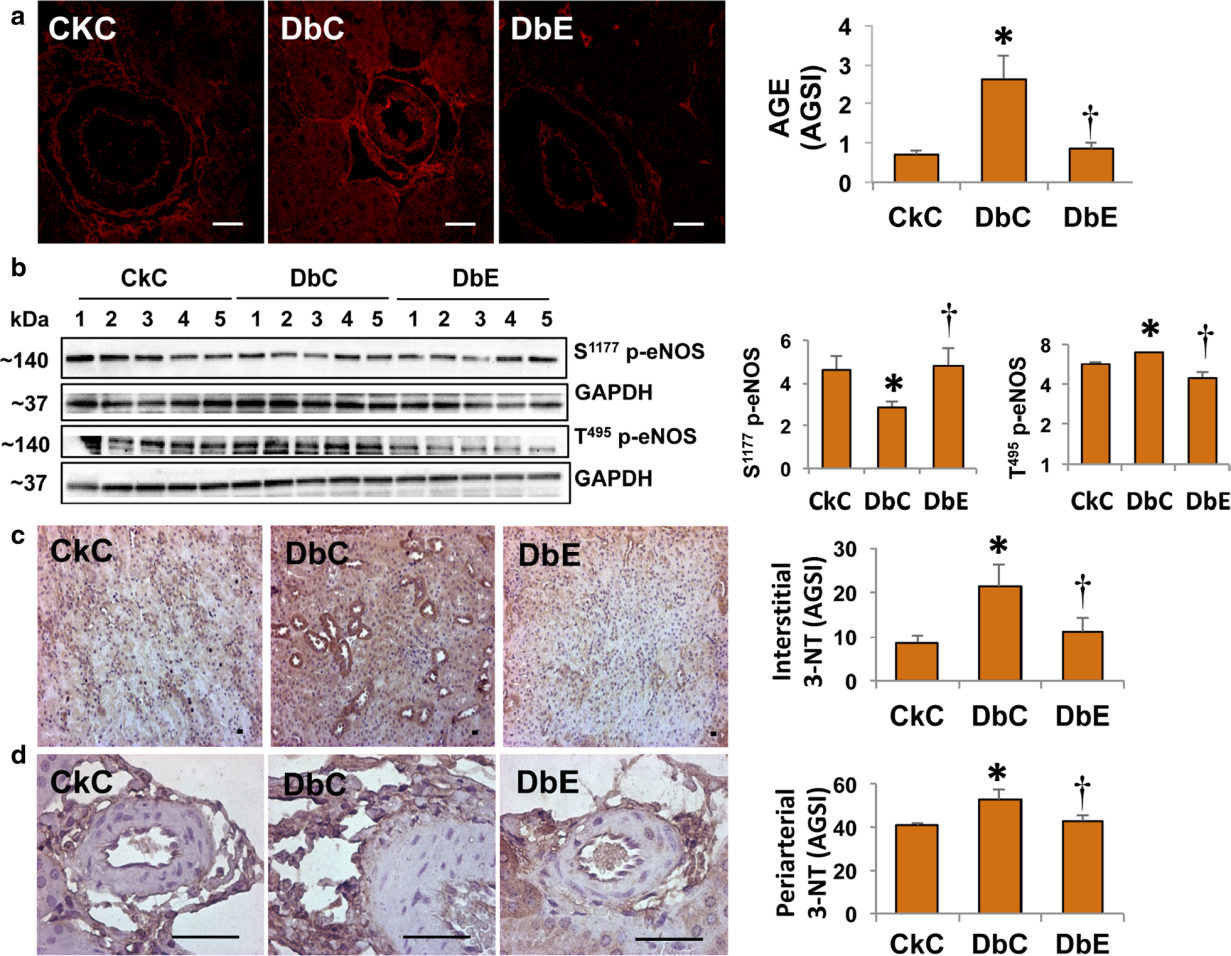
Empagliflozin suppresses advanced glycation end products (AGE), restores endothelial nitric oxide synthase (eNOS) activation and reduces interstitial and periarterial nitroso-oxidative stress. **a** Representative micrographs show AGE expression in renal arterioles, assessed by immunofluorescence labeling. The accompanying bar graphs show a semi-quantitative analysis of average immunofluorescence intensities obtained from five randomly selected arterioles (n = 9–11/group). **b** Western blot analysis shows changes in expression of phospho-eNOS^ser1177^, phospho-eNOS^T495^, and GAPDH. The bar graphs to the right show quantitative analyses of protein expression of these serine (1177) and threonine (495) phosphorylated eNOS proteins normalized to GAPDH. Representative micrographs show 3-nitrotyrosine immunostaining in the (**c**) interstitium and (**d**) periarteriolar regions of the kidney (magnification = 4× and 40×, respectively). The accompanying bar graphs show a semi-quantitative analysis of average immunofluorescence staining intensities obtained from five randomly selected areas (n = 6/group). *p < 0.05 vs. CkC; ^†^*p *< 0.05 vs. DbC. All values are mean ± SE

The presence of AGE has been directly linked to reductions in activation of eNOS and impairments in vessel relaxation and stiffness [42–44]. Our results showed increased formation of AGE, localized primarily in vascular tissue at the periadventitial region in the kidney cortex of DbC compared to CkC (Fig. 4a, p < 0.05). This increase in AGE levels was associated with reduction in S1177 phosphorylation and a concomitant increase in T495 phosphorylation of eNOS in kidney cortical homogenates of DbC, compared to CkC (Fig. 4b, p < 0.05). Further, presence of 3-nitrotyrosine (3-NT), a surrogate of oxidative and nitrosative stress, was increased in the cortex, as well as in the periarteriolar space in the kidneys of DbC, compared to CkC, (Fig. 4c and d, p < 0.05), indicating that EMPA inhibited nitrosative stress in the kidneys of diabetic mice.

### EMPA rescues RECK expression in diabetic kidneys

**Fig. 5:**
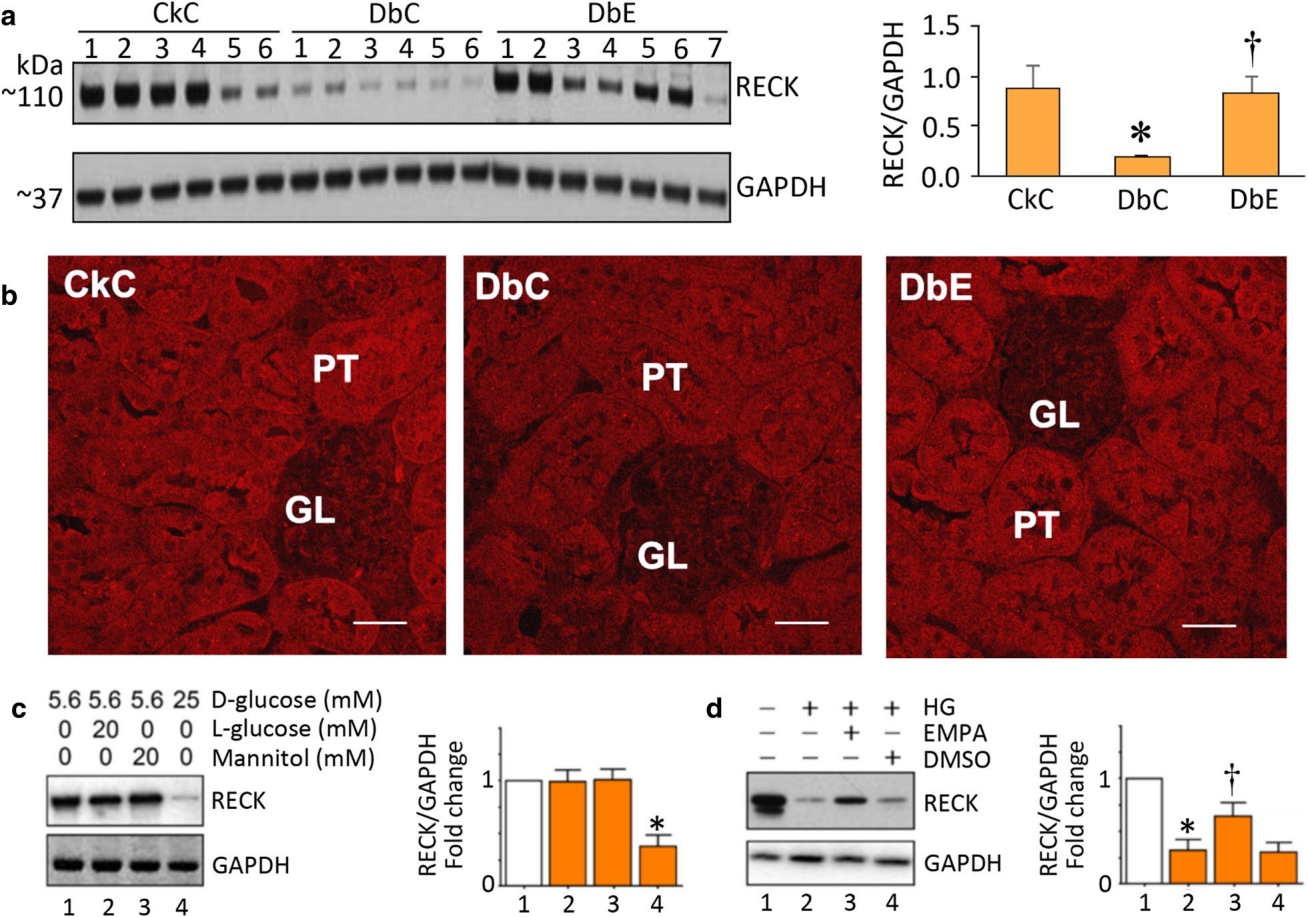
Empagliflozin rescues RECK deficiency in the diabetic kidney and in high glucose (HG)-treated renal proximal tubule cells. **a** Western blot analysis shows changes in RECK expression in kidneys from control (CkC), diabetic (DbC) and EMPA-treated diabetic (DbE) mice. The accompanying bar graph shows quantitative analysis of RECK protein expression as fold change from baseline in the CkC group. *p < 0.05 vs. CkC; ^†^p < 0.05 vs. DbC. **b** Immunofluorescence staining indicates high level of expression in proximal tubule cells and lesser expression in the glomerulus. Magnification = 63× and scale bars = 50 μm. **c**, **d** The SGLT2 inhibitor EMPA restores high glucose (HG)-induced inhibition in RECK expression in cultured human renal proximal tubule epithelial cells (HK-2). High glucose suppresses RECK expression in HK2 cells with mannitol serving as an osmotic control (**c**). EMPA pretreatment reversed HG-induced suppression in RECK expression (**d**). While representative immunoblots from three independent experiments are shown to the left, the intensities of immuno-reactive bands from all three experiments are summarized on the right. The values are mean ± SE. *p < 0.05 vs. low glucose, p < 0.05 vs. high glucose. *p < 0.05 vs. CkC; ^†^p < 0.05 vs. DbC

Compared to CkC, RECK protein expression was significantly suppressed in DbC (Fig. 5a; p < 0.05) and was partially restored by EMPA (p < 0.05). Across all groups, immunofluorescence revealed high levels of RECK expression in cortical proximal tubule cells and low levels in distal tubule cells and glomerulus (Fig. 5b; p < 0.05).

### EMPA reverses high glucose-induced RECK suppression in HK-2 cells

In support of our in vivo evidence of RECK suppression in the diabetic kidney (Fig. 5a, b), we observed that high glucose (25 mM) significantly suppressed RECK expression in HK-2 cells. This suppression induced by high glucose was rescued by EMPA (Fig. 5c, d), suggesting that EMPA is a positive regulator of RECK expression in kidneys.

## Discussion

**Fig. 6:**
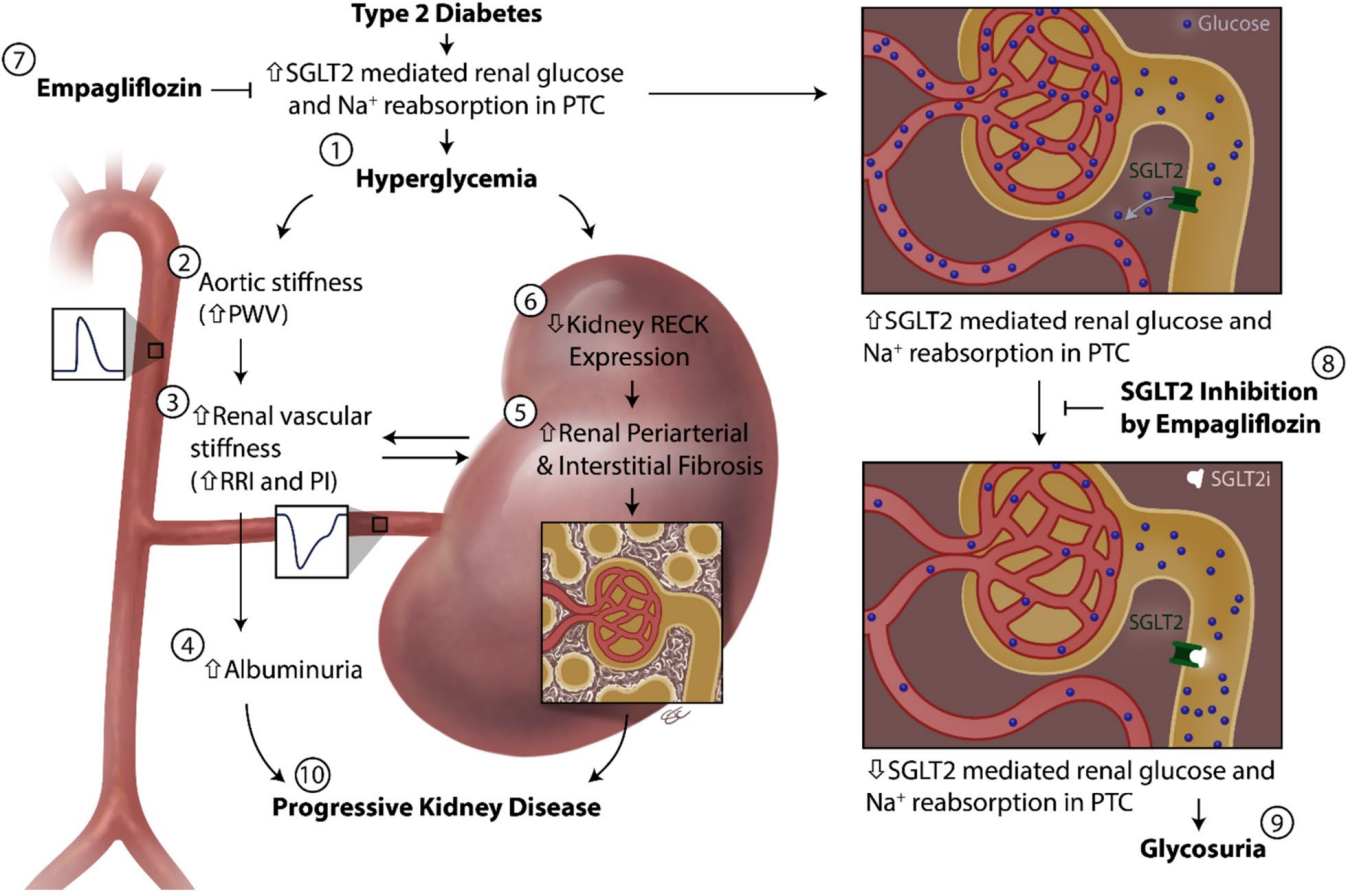
Glycemic control by the SGLT2 inhibitor empagliflozin (EMPA) decreases macro- and micro-vascular stiffening, renal resistivity index, and kidney injury. Hyperglycemia (1) in type 2 diabetes causes aortic stiffness (2), which is associated with increased renal arterial stiffness (3), albuminuria (4) and tubulointerstitial fibrosis (5). Hyperglycemia suppresses the anti-fibrotic factor RECK in proximal tubular cells (6). Importantly, inhibition of SGLT2 by EMPA (7 and 8) enhances glycosuria (9), inhibits aortic and renal vascular stiffening, reverses RECK suppression, reduces kidney fibrosis and albuminuria, and blunts progression of kidney disease (10)

Here we show for the first time that SGLT2 inhibition attenuates diabetes-induced systemic macrovascular and renal microvascular stiffness, as well as kidney injury and fibrosis. The improved glycemic control by EMPA is associated with enhanced renal eNOS activation and suppressed nitrosative stress. Furthermore, EMPA rescued hyperglycemia-induced suppression of the anti-fibrotic factor, RECK, in the diabetic kidney. Collectively, these data suggest that both vascular stiffening and renal fibrosis contribute to progression of diabetic nephropathy which can be mitigated by EMPA (Fig. 6).

The beneficial effect of EMPA on progression of kidney disease in the EMPA-REG OUTCOME TRIAL and pre-clinical studies has been ascribed to local hemodynamic changes, whereby SGLT2 inhibition results in decreased sodium reabsorption with consequent increased sodium delivery to the macula densa, increased afferent arterial tone and decreased estimated glomerular filtration rate (GFR), indicative of reduced hyperfiltration [17, 21, 45, 46]. With EMPA, this initial decrease in GFR stabilizes, and over a period of time, improves compared to control subjects. In addition, the resulting reductions in post-glomerular perfusion would predict an increase in acute kidney injury (when GFR < 60 ml/min), however EMPA suppressed kidney injury [45]. Taken together, the effects unrelated to increased afferent tone might have contributed to the beneficial effects of EMPA in slowing progression of renal injury in diabetic subjects. In this regard, hyperfiltration can be affected by worsening endothelial dysfunction and aortic stiffness [47]. We hypothesized that suppression of macrovascular and microvascular stiffness may also be contributing to amelioration of microalbuminuria due to effective glycemic control by EMPA. The improvement of vascular stiffness, along with improved endothelial function by EMPA in this study may contribute, in part, to hyperfiltration, as well as, direct effects on vascular function in improving microalbuminuria (Fig. 6).

A number of clinical studies support the existence of relationships between increased aortic stiffness, albuminuria, and kidney injury [7–9, 48]. However, the mechanisms behind these interactions are not well understood. In this regard, aortic stiffening has been shown to propagate excessive pulsatile (kinetic) energy into peripheral organs, such as the kidney, where there is high flow but low precapillary resistance [5, 7, 8, 11]. In aging humans, increases in resistivity and pulsatility in the kidney vasculature have been linked to reductions in glomerular filtration rate, albuminuria and chronic kidney disease [7]. However, the application of resistivity and pulsatility indexes to the exploration of mechanisms of renal vascular dysfunction in preclinical models has been limited [7, 9]. In this study, we utilized a novel non-invasive ultrasound technique to evaluate indices of kidney microvascular health (RRI and PI) in a preclinical model of diabetic nephropathy, as has been previously reported [49].

Stiffening of the aorta and microvasculature has been linked to kidney parenchymal injury and fibrosis [50, 51]. In this study, we observed significant increases in periarterial and tubulointerstitial fibrosis with concomitant increases in RRI and PI. Importantly, EMPA abrogated these structural and functional abnormalities. These findings support the notion that increases in cortical tissue periarterial and tubulointerstitial fibrosis, along with aortic stiffness, contribute to renal vascular stiffness and albuminuria in diabetes. One of the pathways that regulates kidney fibrosis is impairment in eNOS-dependent NO signaling [52]. In fact, we observed differential phosphorylation of eNOS (reduced S1177 phosphorylation and increased T495 phosphorylation) favoring its inactivation, and presumably reduced bioavailable NO, in diabetic kidneys. Notably, EMPA treatment restored eNOS activation, possibly contributing to reduced kidney injury and improved renal vascular function. In support of this notion, it has been previously shown that the reno-protective effects of EMPA were lost in type 1 diabetic mice deficient in eNOS [53]. Of note, hyperglycemia and increased AGE levels have also been shown to reduce bioavailable NO [42, 54]. In the present study, we showed that diabetic mice exhibit increased AGE in kidney cortical tissue along with reductions in eNOS activation and increases in periarteriolar and tubulointerstitial fibrosis. Therefore, effective control of hyperglycemia resulting in suppression of AGE expression, decreased nitrosative stress and increased eNOS activation collectively may contribute to improvement in kidney structure and function.

RECK is a membrane-anchored protein that suppresses a pro-fibrotic response through inhibition of MMP activation [30, 31]. However, its role in diabetes-induced renal injury and fibrosis has not been investigated. We previously reported that activation of the RAAS induces cardiac fibrosis with associated suppression in RECK expression in the heart [30]. However, it is not known whether hyperglycemia alone suppresses RECK expression in the kidney. Our data show that normal kidneys express high levels of RECK (Fig. 5a) that is predominantly localized to cortical proximal tubule cells and to a lesser extent in glomerular ECs. Notably, RECK expression was significantly downregulated in diabetic kidneys, but partially restored by EMPA. Supporting these in vivo studies, high glucose suppressed RECK expression in cultured proximal tubule epithelial cells and this suppression was prevented by EMPA. To our knowledge, this is the first report describing RECK downregulation in diabetic kidneys and its rescue by EMPA. Our future studies will determine the molecular mechanisms underlying RECK regulation by hyperglycemia/EMPA.

SGLT2 inhibition improves albuminuria in humans [55] and in preclinical models of diabetes and kidney fibrosis [56, 57]. Similarly, we observed decreased albuminuria and kidney fibrosis in EMPA-treated female diabetic mice. However, in a comparable study using a similar dose of EMPA, no improvement in albuminuria was observed in male db/db mice of similar age, despite similar histopathological changes in the kidney [58]. Interestingly, the magnitude of glycemic control needed to reduce albuminuria appears to be a glucose concentration of 15 mmol/l or less [21]. In the present study, we observed improvement in albuminuria in concert with blood glucose levels below 15 mmol/l. In the other study that employed male mice [58], blood glucose levels remained above 15 mmol/l with no improvement in albuminuria. Thus, these comparable studies performed in male and female db/db mice offer insights into the notion that the magnitude of glycemic control may be an underlying factor for the responsiveness of kidneys to progressive renal injury by SGLT2 inhibition [19].

Aortic and renal vascular stiffness are commonly associated with high BP, and EMPA has been shown to decrease BP and arterial stiffness in humans [28, 29]. However, in our model, reductions in aortic and microvascular stiffness by EMPA were not associated with improvements in BP [40], indicating that additional mechanisms, independent of BP reduction, might have contributed to the reno-protective effects of EMPA. Indeed, our BP results are consistent with one other study reporting no differences in BP following 10 weeks of EMPA treatment in db/db mice [59]. In this regard, our data suggest that reduced EC stiffening and enhanced eNOS activation might have contributed to the beneficial vascular effects of EMPA. In fact, we and others have previously demonstrated an association between impaired vascular NO signaling and arterial stiffening in a diet-induced obesity model, independent of changes in BP [60, 61].

It is noteworthy that despite improved glycemia, EMPA was not associated with improvement in plasma lipids, fat pad mass or body weight. As far as we are aware, EMPA is not reported to lower total cholesterol, free fatty acids or body weight in rodents [33, 56, 58], including db/db mice [58]. However, though EMPA treatment is associated with weight loss in diabetic patients, it either elevated or had no effect on serum lipids in humans (versus placebo) [62–64]. It has also been suggested that there may be species-dependent effects of SGLT2 inhibition on body weight, and that other factors such as activity level and food intake, might contribute to divergent responses in these outcomes [33].

A limitation of the present study is that we did not examine the direct effects of EMPA treatment on cultured ECs or vascular rings. One recent study demonstrated pleiotropic effects of EMPA in cultured ECs [56], while another suggested that EMPA does not affect vasoreactivity of aortic rings from mice or rats [65]. Interestingly, a more recent investigation indicated a robust increase in SGLT1 and SGLT2 expression in rat aortas, as well as high glucose-treated porcine ECs [66]. Thus, it is possible that glycemic control by EMPA, via inhibition of SGLT2 in kidney proximal tubules, as well as in the vascular wall, contributes to improvement in vascular stiffness and kidney function. Further work is needed to elucidate the role of endothelial SGLT2 expression in modulating vascular health. Although recent studies show renoprotection with other SGLT2 inhibitors, suggesting possible class effects [67, 68], the individual underlying drug effects and their specific mechanisms remain to be determined. Further, completion of several ongoing clinical trials, including, EMPEROR-Reduced, EMPEROR-Preserved, DAPA-HF, DAPA-DKD and CREDENCE, will shed insights into the CV and renoprotective effects of SGLT2 inhibitors [68].

In summary, we demonstrate an association between aortic stiffness, endothelial dysfunction, renal vascular stiffness and kidney injury in diabetic mice, that is improved with SGLT2 inhibition by EMPA, independent of changes in BP or body weight. We also report evidence of RECK deficiency in diabetic kidneys, and its rescue by EMPA. Together, these data indicate that glycemic control by EMPA exerts vascular- and reno-protective effects in diabetic mice, resulting in reduced kidney fibrosis and injury.

## Abbreviations

ACEi: angiotensin-converting enzyme inhibitors
ARBs: angiotensin type 1 receptor blockers
DKD: diabetic kidney disease
CVD: cardiovascular disease
RAAS: renin–angiotensin–aldosterone system
SGLT2: sodium-glucose co-transporter 2
EMPA: empagliflozin
RECK: reversion inducing cysteine rich protein with Kazal motifs
CkC: untreated C57BLKS/J mouse
DbC: untreated db/db mouse
DbE: db/db treated with EMPA
RRI: renal resistivity index
PI: pulsatility index
EC: endothelial cell
VSMC: vascular smooth muscle cell
IEL: inner elastic lamina
EL: elastic lamina
N: nucleus
L: lumen
LRA: left renal artery
LDV: lowest diastolic velocity
PSV: peak systolic velocity
CSA: cross sectional area
PWV: pulse wave velocity
cPWV_inc_: calculated incremental PWV
D: luminal diameter
τ: wall thicknesses
${E_{inc}}$: the incremental modulus of elasticity
AFM: atomic force microscopy
GAPDH: glyceraldehyde 3-phosphate dehydrogenase
ColIα1: collagen Iα1
ColIIIα1: collagen IIIα1
FN: fibronectin
AGE: advanced glycation end products
AGSI: average grey scale intensities
eNOS: endothelial nitric oxide synthase
3-NT: 3-nitrotyrosine
PTC: proximal tubule cells
Ach: acetylcholine
MMP: matrix metalloproteinase
HK-2: human proximal tubular epithelial cells
HG: high glucose
DMSO: dimethylsulfoxide
MTT: (3-(4,5-dimethylthiazol-2-yl)-2,5-diphenyltetrazolium bromide)
EMPA-REG OUTCOME TRIAL: empagliflozin, cardiovascular outcomes, and mortality in type 2 diabetes trial
PSS: physiological salt solution
PSR: picrosirius red
